# Two Methods for the Isolation and Cultivation of Porcine Primary Corneal Cells

**DOI:** 10.3390/mps6030050

**Published:** 2023-05-12

**Authors:** Alice Rocha Teixeira Netto, Marc Dieter Hrusa, Karl-Ulrich Bartz-Schmidt, Sven Schnichels, José Hurst

**Affiliations:** Centre for Ophthalmology, Clinical Research University Eye Hospital Tübingen, Eberhard Karls Universität Tübingen, Elfriede-Aulhorn-Straße 7, D-72076 Tübingen, Germany

**Keywords:** cornea, epithelial cells, corneal cells, primary culture, porcine corneas

## Abstract

In ophthalmic research, there is a strong need for in vitro corneal cell models. Here, we describe different protocols for the cultivation of primary corneal cells that were isolated from porcine eyes. This primary cell culture can be used to test new therapeutic options for corneal diseases, such as dry eye disease, traumatic injuries, or corneal infections, and to study limbal epithelial stem cell (LESC) expansion. Two different isolation methods were performed: the outgrowth and the collagenase method. To perform the outgrowth protocol, small explants of the corneal limbus were generated and incubated in culture flasks in an incubator for 4–5 weeks. Regarding the collagenase method, to extract corneal cells, porcine corneas were removed, cut into small pieces, and incubated with collagenase. After incubation and centrifugation, the cells were seeded in 6- or 12-well plates and incubated in an incubator for 2–3 weeks. The differences between corneal cell cultivation with fetal bovine serum (FBS) and without it are also discussed. Therefore, the main advantages of the outgrowth method are that it requires fewer porcine eyes, and it takes less time to be performed compared to the collagenase method. On the other hand, with the collagenase method, mature cells are obtained earlier, at about 2 to 3 weeks.

## 1. Introduction

The cornea is a transparent avascular structure and a structural barrier of the eye [1]. It is also an important refractive component of the visual system being responsible for approximately 70% of the total refractive power of the eye [2]. The external layer of the cornea is the epithelium, which is a stratified, non-keratinizing layer, and it can be composed of up to seven cell layers. The most external layer of the corneal epithelium is formed from flat polygonal cells with microvilli and microplicae, which are covered by the tear film [3]. The middle layer of the corneal epithelium is constituted by two or three layers of wing cells. The most internal corneal epithelium layer is the basal layer, which is made up of a single layer of columnar epithelial cells [1]. The middle layer of the cornea, the stroma, is the thickest one, constituting around 90% of the cornea, and it is made up of a stromal matrix of collagen fibrils and keratocytes [4]. The posterior layer of the cornea is a single layer of cells called endothelium, where the endothelial cells are responsible for keeping the transparency of the cornea by regulating the circulation of aqueous humor into the cornea [5].

The corneal epithelial cells express cytokeratins, such as cytokeratin 3 (CK3) and cytokeratin 12 (CK12), and are rich in tight junctions that are constituted of zonula occludens 1 (ZO-1) and occludin, among other proteins [6,7]. Vimentin is a member of intermediate filament type III, which is part of the cytoskeleton. It can be expressed in a basal manner by keratocytes and also by fibroblasts and myofibroblasts [8]. The undifferentiated epithelial cells localized at the proliferative rim of the cornea also express vimentin [9]. The keratocytes in the stroma synthesize keratan-sulfate-containing proteoglycans such as keratocan and lumican [10]. When the corneal epithelial barrier is breached and the stroma is exposed, corneal keratocytes become activated and differentiate into fibroblasts [11].

Corneal diseases are considered an important cause of blindness in the world. It is estimated that 6.7 million people worldwide have visual impairment associated with corneal diseases [12]. The cornea can be affected by several diseases and injuries, such as ulcerative lesions, keratoconus, leukoma, bullous keratopathy, dystrophies (with the most common being Fuchs’), infections, inflammations, and perforations, among many others. These diseases lead to opacification, moderate or severe visual impairment, and even blindness [13].

Limbal insufficiency, which can occur due to frequent ocular diseases, for example, chemical burns or unusual ocular diseases, such as ocular cicatricial pemphigoid, constitutes a therapeutic challenge. Without the regenerative function of the limbal region, the cornea will not be able to renew its cell layers [14,15]. Almost every cell type of the cornea finds its origin in stem cells of this particular area. When limbal insufficiency occurs, conjunctival epithelial ingrowth, inflammation, and destruction of the basal membrane lead to visual impairment. Because of that, limbal epithelial stem cell (LESC) therapy is a feasible alternative treatment option for limbal insufficiency [16]. Hence, methods for expansion of limbal epithelial stem cells (LESCs) ex vivo are required. LESCs are located at the “palisades of Vogt”, and their proliferation originates corneal epithelial cells [17].

Moreover, corneal limbal isolation and primary corneal cell cultivation are also useful to produce an in vitro cell model to test new therapeutic options for corneal diseases, such as corneal infection or dry eye disease. Since a high number of animals is used for research purposes, alternatives to the use of laboratory animals are required due to ethical reasons [18]. So, another big advantage of both methods described is that the porcine eyes used were not taken from animals that were just used for research grounds. As we described previously, the porcine eyes used for research were received from a local abattoir, and the animals were slaughtered for nutritional purposes, so the euthanasia of animals only for scientific purposes is avoided [19].

Besides this important advantage, it has been demonstrated that porcine eyes have a similar morphology to the human eye, and the pig is phylogenetically close to the human [20,21]. Furthermore, human tissues are difficult to obtain, so animal tissues are often used as a substitute. Finally, obtaining porcine eyes from an abattoir is less expensive than breeding animals for scientific research only. For these reasons, porcine eyes were chosen for the development of these isolation methods.

Here, we describe and compare two different methods of isolation of porcine corneal cells: the outgrowth and the collagenase method. Isolating cells with the outgrowth method is a faster, taking about 2 h, and requires a lower number of porcine eyes compared to the collagenase one. Moreover, it is easier to change the culture medium in the weeks after isolation, once the cells are growing in a flask. However, for the cells to reach 70–80% confluence, it takes 4–5 weeks. On the other hand, with the collagenase method, mature cells are obtained earlier, at about 2 to 3 weeks.

## 2. Experimental Design

The protocols describe primary corneal cell culture, extracted from porcine corneas, cultivated in a specific epithelial cell medium. These protocols are simple to perform, easy to standardize, and avoid the use of animals exclusively for research purposes. Porcine eyes were collected from a local abattoir, where the animals are euthanized for nourishment.

After disinfection, the corneas were extracted under sterile conditions. Two different methods of cell isolation and cultivation were performed: the outgrowth and the collagenase method. To perform the outgrowth protocol, the whole corneal limbus was dissected from the remaining corneal tissue and cut into smaller explants. After placing 15–20 explants with culture medium in each culture flask, they were incubated in a sterile incubator at 37 °C with 5% CO_2_ for 4–5 weeks. 

To extract epithelial cells with collagenase, porcine corneas were removed, cut into small pieces, and incubated with collagenase. After incubation and centrifugation, the cells were seeded in 12-well plates or 6-well plates and incubated in an incubator at 37 °C with 5% CO_2_ for 2–3 weeks.

It is important to highlight that, with these methods, more than one corneal cell type is cultured, so a pure culture of corneal epithelial cells cannot be obtained. Another limitation of these methods is a greater chance of contamination since the cells have been isolated from porcine eyes. Therefore, the entire cell isolation procedure must be performed under strictly sterile conditions to keep the chances of contamination at a minimum. Furthermore, it is important to notice that although pig eyes are very similar to human eyes [20], differences between species exist.

### 2.1. Materials

1. Bovine serum albumin (BSA) (PanReac AppliChem ITW Reagent, Darmstadt, Germany; Cat. No. A1391,0500).
2. 8-well chamber slides (Thermo Scientific™ Nunc™ Lab-TekII™, Rochester, NY, USA; Cat. No. 154534).
3. CnT-Prime (CellnTec-Advanced Cell Systems, Bern, Switzerland; Cat. No. CnT-PR)
4. Collagenase 100 mg (0.171 U/mg lyo.) (Sigma-Aldrich, Taufkirchen, Germany; Cat. No. 10103578001).
5. Culture flask with ventilation screw cap T25 (Greiner Bio-One Gmbh, Frickenhausen, Germany; Cat. No. 690175).
6. Disposable cup 100 mL (Sarstedt, Nümbrecht, Germany; Cat. No. 75.563).
7. Disposable safety scalpel #11 (Aesculap AG, Tuttlingen, Germany; Cat. No. BA811SU).
8. Dulbecco’s Phosphate Buffered Saline (PBS) (Gibco™ Thermo-Fisher, Karlsruhe, Germany; Cat. No. 10010056).
9. Eppendorf Safe-Lock tube 2 mL (Eppendorf, Hamburg, Germany; Cat. No. 0030121880).
10. Ethanol 70% (VWR, Darmstadt, Germany; Cat. No. 97065-058).
11. Tube 15 mL (Greiner Bio-One, Frickenhausen, Germany; Cat. No. 188271).
12. Fetal bovine serum (FBS) (Thermo-Fischer, Karlsruhe, Germany; Cat. No. 10100147).
13. FluorSave™ (Merck Milipore, Darmstadt, Germany; Cat. No. 345789).
14. Iodine (Braunol^®^, B. Braun, Melsungen, Germany; Cat. No. 190970).
15. MultiMACS cDNA Synthesis Kit (Miltenyi Biotec, Köln, Germany; Cat. No. 130-094-410).
16. Paraformaldehyde (PFA) (Merck Milipore, Darmstadt, Germany; Cat No. 1040051000).
17. Penicillin–streptomycin 10,000 U/mL (Thermo-Fischer, Karlsruhe, Germany; Cat. No. 15140148).
18. iTaq Universal SYBR^®^ Green Supermix (Bio-Rad, Feldkirchen, Germany; Cat. No. 1725124).
19. Trypsin/EDTA (Gibco™ Thermo-Fischer, Karlsruhe, Germany; Cat. No. 25200072).
20. Triton X-100 (Sigma-Aldrich, Taulfkirchen, Germany; Cat. No. 9002-93-1).
21. 6-well plate (Greiner Cellstar^®^, Frickenhausen, Germany; Cat. No. M9062-100EA).
22. 12-well plate (Nerbe plus, Winsen/Luhe, Germany; Cat. No. 14-020-0012).
23. 4′,6-diamidino-2-phenylindole (DAPI) (Thermo-Fischer, Karlsruhe, Germany; Cat. No. D1306).

### 2.2. Equipment

- Olympus R1 SLI Cell counter (Olympus coorporation, Tokyo, Japan; Cat. No. K23009240).
- Centrifuge VWR Mega Star 3.0R (VWR, Darmstadt, Germany; Cat. No. 521.1752).
- Heracell 150i CO_2_ incubator (Thermo-Fischer, Karlsruhe, Germany; Cat. No. 50116050).
- MultiMACS™ M96 Separator (Miltenyi Biotec, Köln, Germany; Cat. No. 130-091-937).
- VWR Thermal Shake Touch (VWR, Darmstadt, Germany; Cat. No. 89232-908).
- Thermal cycler Bio-Rad CFX96™ Real-Time System (Bio-Rad, Feldkirchen, Germany; Cat. No. 1845097).
- Zeiss Axio Observer (CarlZeiss, Oberkochen, Germany; Cat. No. 491916-0002-000).
- Zeiss Axio Imager Z1 Apotome Microscope with Mrm digital camera (CarlZeiss, Oberkochen, Germany; Cat. No. p1739).

## 3. Procedure

### 3.1. Corneal Cell Culture and Splitting

#### 3.1.1. Checklist (6 h)

1. To guarantee sterile conditions and avoid contaminations, clean and disinfect the laminar-air-flow hood with 70% ethanol.
2. Disinfect and autoclave tools (e.g., scissors and forceps) before use.
3. Prepare the following medium in advance under a laminar-air-flow hood, under sterile conditions: CnT-Prime, 2% penicillin–streptomycin. Fetal bovine serum (FBS) can be added in different concentrations (e.g., 10%).
4. Adjust all substances to room temperature before use.

#### 3.1.2. Corneal Cell Outgrowth Protocol (2 h)

**Figure 1:**
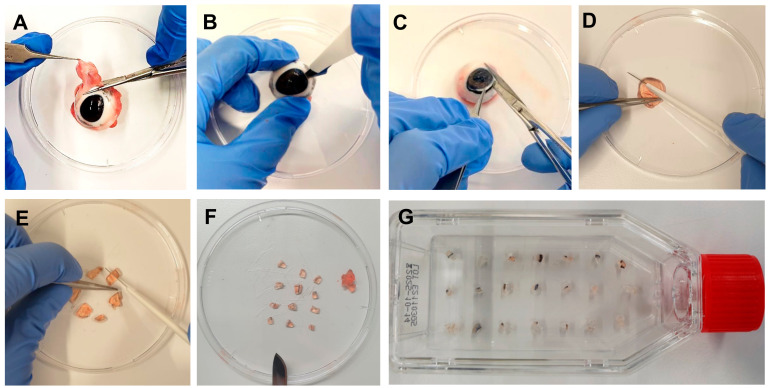
Step-by-step corneal cell outgrowth method. (**A**) Muscles and tissues around the eye were removed. (**B**,**C**) To extract the cornea, an incision was made in the sclera with a scalpel, and the cornea was finally cut out with scissors. (**D**) The cornea was cut in half. (**E**,**F**) Each cornea half was cut in pieces, and the center of the cornea was discarded, so that a strip of approximately 2 mm of corneal tissue and 2 mm of sclera was produced. (**G**) The small cornea explants were placed in a culture flask with a ventilator screw cap. After waiting for 5 min until the cornea explants adhered to the surface of the flask, 1 mL of culture medium was added (CnT-Prime + 2% PS), one drop per explant. Then, 10% FBS was added to the medium, depending on the purpose of the culture.

1. Collect enucleated porcine eyes from a local abattoir just after sacrification of the animals and store them on ice during transportation to the laboratory. Avoid direct contact between the eyes and the ice. At the laboratory, store the eyes at 4 °C until use. Process the eyes as soon as possible. The longer the storage at 4 °C, the lower the tissue quality.
2. Remove the eyes from the refrigerator and remove muscles and tissues around the eye with scissors and forceps (Figure 1A). Do not harm the eyeball. One eye is needed to isolate explants before sufficient cultivation of one flask.
3. Disinfect the eye in iodine 5% (*v/v*) diluted in distilled water for 5 min in a disposable cup.
4. Wash the eye 5 times in phosphate-buffered saline (PBS) with 2% penicillin-streptomycin (PS).
5. In a petri dish, extract the pig cornea from the eye with a scalpel, scissors, and forceps by cutting circularly around the cornea, leaving a thin part of the sclera around it (Figure 1B,C).
6. Discard the rest of the eye, clean the cornea in PBS with PS, first cut it in half, and then cut each half into three parts. Extract the center of the cornea, leaving a strip of approximately 2 mm of corneal tissue and 2 mm of sclera (Figure 1D,F).
7. Place the explants of one eye in a T25 culture flask with a ventilation screw cap (Figure 1G). Use 15–20 explants per culture flask (cells grow most effectively when they are closer to other corneal explants).
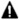
**CRITICAL STEP:** Wait 5 min until the cornea explants adhere to the surface of the flask and carefully add 1 mL of culture medium (CnT-Prime + 2% PS), one drop per explant, so that the explants do not detach from the flask. Only attached explants assure outgrowth. Floating explants should be removed. 10% FBS can opitionally be added to the culture medium.
9. Incubate the flask in an incubator at 37 °C with 5% CO_2_ overnight.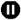 PAUSE STEP
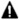
**CRITICAL STEP:** On the next day, carefully add 3 mL of culture medium to the flask. Pay attention and make sure that the explants do not move.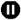 PAUSE STEP
11. Incubate the flask in an incubator at 37 °C with 5% CO_2_ for 4–5 weeks and change the culture medium twice weekly. To change the medium, aspirate carefully the old medium to avoid detachment of corneal explants and slowly add 4 mL of new culture medium.

The cells will achieve 70% confluency in approximately four weeks. Depending on the aim of the envisaged experiment, the cells can be split in different sizes of well plates or in chamber slides.

#### 3.1.3. Splitting Cells (30 min)

1. Remove the corneal explants from the culture flask with the help of forceps.
2. Remove the culture medium with a Pasteur pipette and rinse with 4 mL of PBS.
3. Remove PBS and add 1 mL of trypsin/EDTA (T/E), and then incubate the flask in an incubator at 37 °C with 5% CO_2_ for 5 min.
4. Mechanically detach the cells by tapping on the side of the bottle.
5. Add 2 mL of T/E-stopping solution (CnT-Prime + 2% PS + 10% FBS).
6. Pipette the entire content of the flask into a 15 mL tube and centrifuge it at room temperature, 300× *g* for 5 min.
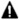
**CRITICAL STEP:** Carefully aspirate the supernatant and resuspend the cell content with 1 mL of culture medium (CnT-Prime + 2% PS with or without FBS depending on whether the explants were previously incubated with serum or not).
8. Count cells, dilute the cells accordingly, and seed them in well plates. The cell numbers are defined according to the area inside the culture vessel (Table 1).

#### 3.1.4. Corneal Cell Extraction with Collagenase Protocol (5 h)

**Figure 2:**
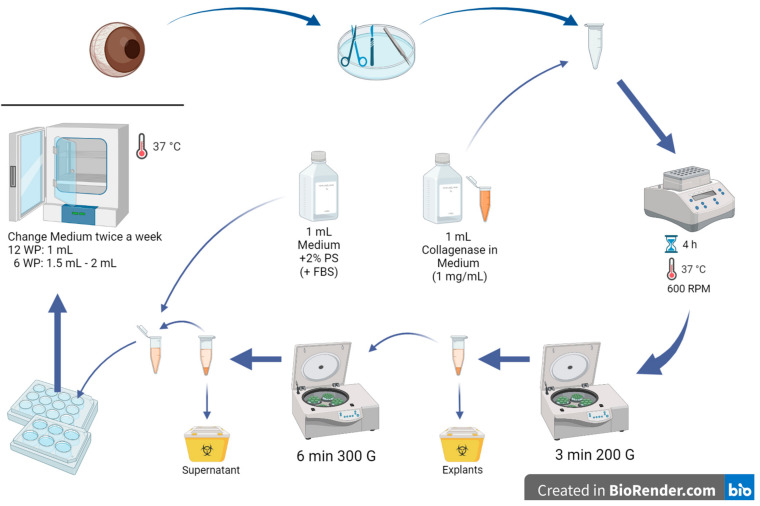
Step-by-step corneal collagenase method. Porcine eyes were received from a local abattoir, muscles and tissues around the eye were removed, and the corneas were extracted. The corneas were cut into small pieces and incubated with medium and collagenase in Eppendorf tubes on a shaker for 4 h. After centrifuging the tubes, the supernatant was transferred to another Eppendorf, and the corneal pieces were discarded. The tube was centrifuged again, and the supernatant was disposed. Then, 1 mL of culture medium was added to the cells remaining at the bottom of the tube, the cells were resuspended, seeded in well plates, and incubated at 37 °C and 5% CO_2_. WP: well plate. Created in BioRender.com.

1. Collect enucleated porcine eyes from a local abattoir just after the sacrifice of the animals and store them in a plastic bag in a foam box refrigerated with ice during the transportation to the laboratory. Do not place the eyes directly onto the ice. At the laboratory, store the eyes at 4 °C until use. Make sure that the eyes are used as soon as possible. The longer the storage at 4 °C, the lower the tissue quality.
2. Select at least 2 and up to 10 pig eyes with intact corneas.
3. Remove the eyes from the refrigerator and discard muscles and tissue around the eye with scissors and forceps. Do not harm the eyeballs.
4. Disinfect the eye with iodine 5% (*v/v*) diluted in distilled water for 5 min in a disposable cup.
5. Wash the eye 5 min in PBS with 2% PS.
6. In a petri dish, extract the pig cornea from the eye with a scalpel, scissors, and forceps by cutting circularly around the cornea, leaving a thin part of the sclera around it (Figure 2).
7. Cut the corneas into small pieces and put them in a 2 mL Eppendorf tube (2–3 pig corneas per tube); make sure to clean your instruments with 70% alcohol from time to time.
8. Add 1 mL of collagenase with a concentration of 1 mg/mL in medium to the tube with cornea explants.
9. Place the Eppendorf tubes on a shaker for 4 h (37 °C, 600 RPM).
10. Centrifuge the tubes for 3 min at 20 °C and 200✕ *g*.
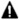
**CRITICAL STEP:** Under the hood, remove the supernatant (with the diluted cells in it) and transfer it into another Eppendorf tube. Dispose of the explants.
12. Centrifuge the tubes again for 6 min at 20 °C and 300✕ *g*.
13. Remove the supernatant and dispose of it.
14. Add 1 mL medium (CnT-Prime + 2% PS) to the remaining cells in the Eppendorf tube. 10% FBS can opitionally be added to the culture medium.
15. Sow the extracted epithelium cells in a 6- or 12-well plate (content of one Eppendorf tube per well) and incubate the well plate in an incubator at 37 °C with 5% CO_2_ overnight. Advice: 12-well plates work better than 6-well plates because the cells may achieve more cell-cell contact in a smaller area (6-well plates may be too big in area for only 2–3 eyes per well).

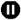
 **PAUSE STEP.**
17. Change the medium twice a week.

#### 3.1.5. Splitting Cells (30 min)

1. Remove the culture medium with a Pasteur pipette, pump, and then rinse with 500 µL of PBS.
2. Remove PBS and add 200 µL of trypsin/EDTA, incubate the 12-well plate in an incubator at 37 °C with 5% CO_2_ for 3–5 min.
3. Mechanically detach the cells by tapping on the side of the well plate.
4. Add the double amount (400 µL) of T/E-stopping solution (CnT-Prime + 2% PS + 10% FBS).
5. Pipette the entire content of the well plate into a 15 mL tube and centrifuge it at room temperature, 300× *g* for 5 min.
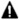
**CRITICAL STEP:** Carefully aspirate the supernatant and resuspend the cell content with 1 mL of culture medium (CnT-Prime + 2% PS with or without FBS depending on whether the explants were incubated with serum or not previously).
7. Count cells with an automated cell counter and add the quantity according to the envisaged experiment (Table 1).

### 3.2. Immunostaining

To confirm the cultivation of corneal epithelial cells, the expression of cytokeratin-3 (CK3), an epithelial cell marker, was analyzed. Moreover, antibodies for zonula occludens 1 (ZO-1) and occludin were used to investigate the presence of tight junctions. To verify the presence of keratocytes in the cultures, keratocan and lumican antibodies were used. To analyze the presence of mesenchymal cells and undifferentiated epithelial cells in the cultures, a vimentin antibody was applied. Cells at the first passage were seeded in chamber slides and were first fixed in 4% Paraformaldehyde (PFA) for 10 min and then washed 4 times with PBS for 3 min. The cells were permeabilized with 0.1% Triton X-100 in PBS for 5 min at room temperature. Afterwards, they were blocked for 1 h with blocking solution (5% (*w/v*) bovine serum albumin (BSA) in PBS). After 1 h, the primary antibody was diluted in 5% (*w/v*) BSA in PBS, according to the concentrations in Table 2, at 5% (*w/v*) BSA in PBS, and then it was pipetted into the samples. The samples were incubated on a shaker overnight at 4 °C. After the incubation, the wells were washed 3 times for 5 min each with PBS. Next, 100 μL of secondary antibody diluted in 5% (*w/v*) in PBS (Table 2) was pipetted on the slides and incubated at room temperature for 1 h in darkness. The slides were then washed 3 times with PBS for 5 min, and 1 μg/mL of 4′,6-diamidino-2-phenylindole (DAPI) was applied to all sections for 5 min. Finally, the slides were washed with PBS. After drying in air, samples were embedded with FluorSave™. The results are described in the Supplementary Materials.

### 3.3. Cell Quantification

Epithelial cells and keratocytes were quantified at the cell cultures. A cell count was performed with semi-automated counter software ImageJ™ (Version 2.0.0-rc-68/1.52e); the total number of cells per picture was quantified; and CK3-, lumican-, and keratocan-positive cells were quantified. The ratio of CK3-positive cells to total cells was calculated to obtain the percentage of epithelial cells. The same procedure was performed to obtain the percentage of keratocytes, and an average between lumican- and keratocan-positive cells was calculated. A minimum of 5 pictures per staining and per condition was used for analysis. The results are described in the Supplementary Materials.

### 3.4. Quantitative Real-Time PCR

The mRNA expressions of the epithelial cell marker *cytokeratin 12* (*CK12*) and the keratocyte marker *keratocan* were analyzed (Table 3). Briefly, after cultivation, the cell cultures were split, and 200,000 cells per Eppendorf tube were frozen in 900 μL of Lysis Buffer. The mRNA was isolated from cell cultures and reverse transcribed using the MultiMACS mRNA, and cDNA synthesis was performed with a cDNA Synthesis Kit on the MultiMACS™ M96 Separator according to the manufacturer’s protocol. After cDNA synthesis, a quantitative real-time PCR (qRT-PCR) was performed using the iTaq Universal SYBR ^®^ Green Supermix in a thermal cycler (Bio-Rad CFX96™ Real-Time System), as described previously [18]. *β-actin* (*ACTB*) and *glyceraldehyde 3-phosphate dehydrogenase* (*GAPDH*) were used as housekeeping genes (Table 3). The results are presented in the Supplementary Materials.

### 3.5. Statistical Analysis 

A statistical analysis was conducted with GraphPad Prism™ Version 8.2.1 (GraphPad Software, San Diego, CA, USA). Student’s *t*-test was performed to compare two groups, after normality was confirmed. Data are presented as mean ± standard error of the mean (SEM). A *p*-value < 0.05 was considered to be statistically significant. The level of significance was set to * *p* < 0.05, ** *p* < 0.01, and *** *p* < 0.001.

## 4. Expected Results

### 4.1. Outgrowth and Collagenase Methods

**Figure 3:**
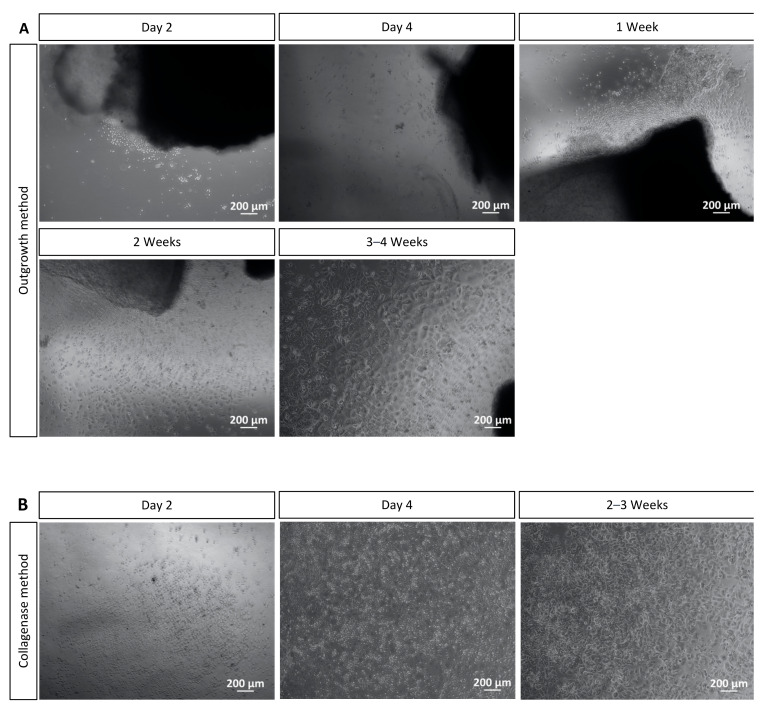
Corneal cell cultivation with the outgrowth and the collagenase methods. (**A**) Cell outgrowth was visualized from corneal explants two days after corneal isolation. After 3–4 weeks of cultivation, most cells were morphologically like epithelial cells and some like keratocytes. After 4 to 5 weeks, cultures achieved 70–80% confluence and could be split. (**B**) Collagenase method: After two days of isolation, the cells attached to the surface of the well plate. After two weeks, homogenous cells, which resemble corneal epithelial cells and some stellate cells, similar to keratocytes, were present. Cell cultures were 70–80% confluent after 2–3 weeks of cultivation. Scale bar = 200 μm.

After isolation of the corneal limbal explants for the outgrowth method, the growth of corneal cells cultured without FBS was observed with microscopy. Cell growth from corneal explants was observed as early as two days after isolation of explants. The cell culture presented homogenous cells with characteristics of corneal epithelial cells and some stellate cells with characteristics of keratocytes (Figure 3A). In this method, the cell cultures reached 70–80% confluency in 4 to 5 weeks of incubation. At this point, the cultures could be split to well plates or chamber slides according to the experiment planned. 

After isolation of corneal epithelial cells with collagenase, the growth of the cells was also detected with microscopy. From two days of corneal epithelial cells’ isolation onwards, cell attachment to the surface of the 12- or 6-well plate was observed, and after four days, a cell differentiation was noticed. The differentiation continued in the following 2–3 weeks. The cells reached 70–80% confluency in 2 to 3 weeks of incubation. At this point, the cultures could be split to well plates or chamber slides according to the envisaged experiment. Again, the culture presented more homogenous cells with characteristics of corneal epithelial cells and some cells with stellate morphology that resemble keratocytes (Figure 3B). 

The biggest advantage of the collagenase method is a quick gain of mature cells in a short time period. However, since there is no intact limbal region anymore, the amount of cell proliferation is limited earlier in comparison to the outgrowth method. Another disadvantage of this method is that the number of porcine corneas needed to perform is larger than the amount used for the outgrowth method. Two corneas are needed for one well of a 12-well plate (Table 4). 

Once the cells are confluent, the collagenase protocol can be used to study wound healing, for example, one could directly make scratches at the center of the wells and evaluate the re-epithelization of these areas, without the need of splitting the cells from the well plates. This method is known as scratch assay [22,23].

Regarding the outgrowth method, it is important to point out that this method can be useful for studying diseases that lead to limbal insufficiency, because the corneal explants have a high number of limbal epithelial stem cells (LESCs).

### 4.2. Cell Cultures Cultivated with Serum Present Higher Amount of Cells

In case a cell culture with higher number of cells, predominantly epithelial cells and fibroblasts, is needed, fetal bovine serum (FBS) 10% should be added to the culture medium. On the other hand, when a cell culture containing primarily epithelial cells and keratocytes is required, FBS should not be included [24].

**Figure 4:**
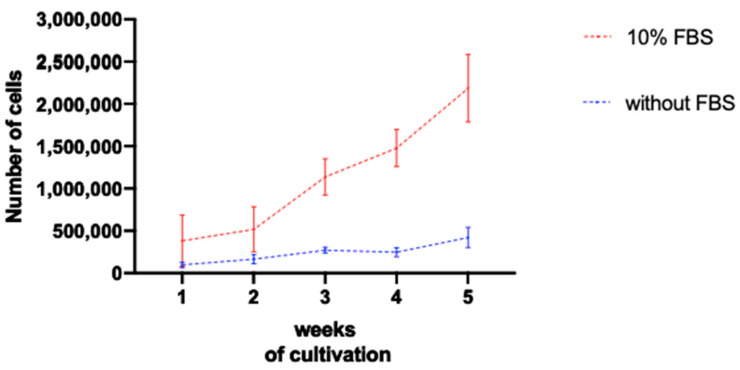
Average number of cells at each week of cultivation with the outgrowth method. The graph confirmed that the number of cells in the cultures incubated with FBS was higher than in the ones without serum. The increase in the number of cells was substantially greater in FBS-containing cultures. The mean values and the SEM are shown.

To evaluate the quantity of cells in each cell culture, with or without FBS, at the outgrowth method, an automated cell counter was used to quantify the number of cells per T25 flask. Each flask was a result of the cultivation of corneal explants coming from one porcine eye. A minimum of three eyes per time point were considered (Figure 4). Cultures incubated with bovine serum showed, since the first week, a higher number of cells that increased even more after two weeks. After five weeks, the number of counted cells cultivated with serum was more than five times higher compared to the group without serum. Therefore, if a high number of corneal cells is needed for an envisaged experiment, the use of FBS is recommended in the culture medium. On the other hand, the cells cultivated without serum increased their number of cells very timidly. This fact is in accordance with the observations made by microscopy and described here.

**Figure 5:**
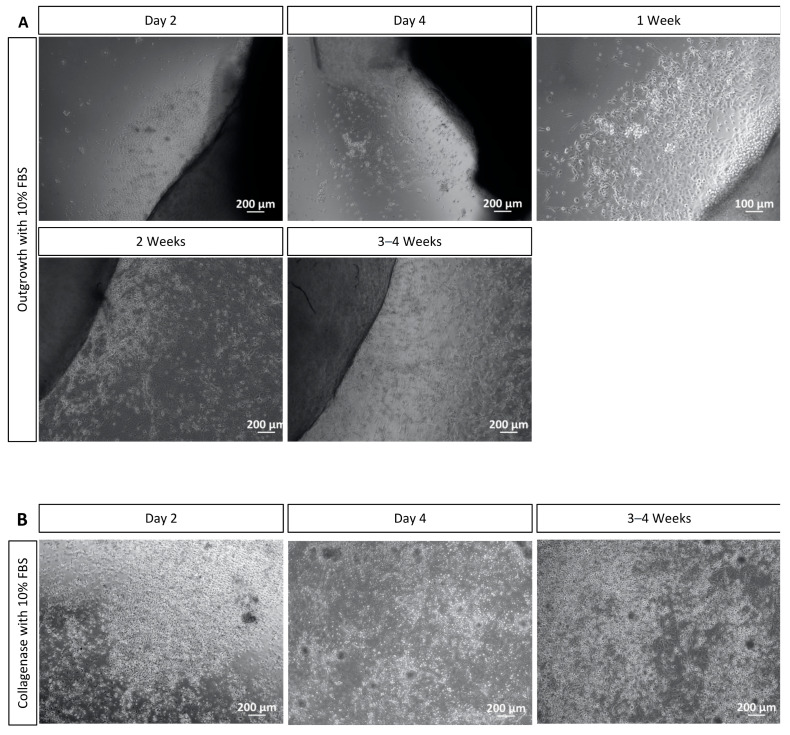
Corneal cell isolation with the outgrowth and the collagenase methods and the use of 10% FBS. (**A**) Cells were observed to grow from corneal explants after two days of isolation. More cells were growing in the serum culture from day 4 until 2 weeks, and some cells resembled fibroblasts morphologically. After 4 to 5 weeks, the culture achieved 70–80% confluence and could be split. (**B**) After two days of isolation, the cells attached to the surface of the well plate. A higher number of cells in the culture cultivated with serum was noticed, and some cells resembled fibroblasts. The cell culture was 70–80% confluent after 2–3 weeks of cultivation. Scale bar = 100 μm and 200 μm.

Regarding the use of FBS in the culture medium at the outgrowth method, cell growth from corneal explants was again observed as early as two days after the isolation of explants. At this stage, there were more cells growing out of the explants cultured with FBS (Figure 5A), and the area of outgrowing cells around the explants was significantly shorter in cultures without FBS (Figure 3A). On the fourth day of cultivation, the largest number of cells growing around the explants cultivated with FBS was observed. This difference could also be noticed in the following two weeks. After two weeks of incubation, the first differences in cell morphology were observed. Cells incubated with 10% FBS were smaller, and there was a higher number of cells at these cultures. During the next weeks, some cells did not grow when it came to cell body size but formed fibrous-like structures (Figure 5A). Therefore, the culture cultivated with FBS also presented some cells morphologically similar to fibroblasts and some epithelial cells (Figure 5A). Again, the cell cultures reached 70–80% confluency in 4 to 5 weeks of incubation. At this point, the cultures could be split to well plates or chamber slides according to the experiment planned.

After isolation of corneal cells with collagenase, we observed cell attachment to the surface of the 12- or 6-well plate from two days of corneal epithelial cells’ isolation onwards. Moreover, it was already possible to notice a higher number of cells when they were cultivated with 10% FBS (Figure 5B). After four days, a higher cell differentiation in this cultivation group was noticed, and this differentiation continued in the following 2–3 weeks. Again, it could be perceived that the culture cultivated with FBS also presented cells that were morphologically similar to fibroblasts (Figure 5B). The cells reached 70–80% confluency in 2 to 3 weeks of incubation. At this point, the cultures could be split to well plates or chamber slides according to the envisaged experiment. 

The main disadvantage of cultivating corneal cells without using FBS is that corneal cells cultivated without FBS take long to grow and are few in number. However, an advantage is the lower number of fibroblasts and a higher occurrence of epithelial cells and keratocytes per eye in this culture. To simulate healthy corneal tissue conditions, the outcome of cells cultivated without FBS is more suited, because a normal corneal stroma has more keratocytes, which are activated and differentiated into fibroblasts in the case of corneal injury. To simulate scaring processes in corneal tissue, a high number of fibroblasts are needed, so the use of FBS in cell cultivation is indicated.

Furthermore, immunostaining and qRT-PCR were conducted to compare cultivation methods with 10% FBS with those without FBS in order to determine the different cell types.

To confirm the cultivation of corneal epithelial cells, the expressions of the epithelial cell marker, cytokeratin-3 (CK3), and the tight junctions’ markers zonula occludens 1 (ZO-1) and occludin were investigated. The corneal epithelium also contains a significant amount of cytokeratin-12 (CK12) [25], so to quantify the mRNA expression of *CK12*, qRT-PCR was performed. Cells from both cultures, with FBS and without it, expressed CK3 (Supplementary Figure S1A). This confirms the hypothesis that both cultures allowed the growth of epithelial cells. Moreover, cells from both cultures expressed occludin and ZO-1 (Supplementary Figure S1A). 

The mRNA expression of *CK12* was also investigated in the cell cultures. The analysis was performed while considering the culture without FBS (1.23 ± 0.42) as the control group, so an upregulation of *CK12* gene expression was observed at the culture cultivated with FBS (4.12 ± 0.88; *p* = 0.02; see Supplementary Figure S1B). This result indicates that the culture cultivated with FBS presented more cells that expressed the *CK12* gene.

The corneal stroma is mainly composed of keratocytes, which are dendritic-shaped cells and produce collagens I, V, VI, and XII and keratan sulfate proteoglycans [10]. These keratan sulfate proteoglycans, such as keratocan and lumican, are important to maintain the transparency of the cornea [26]. To analyze the number of keratocytes in the cell cultures, antibodies targeting keratocyte’s markers, keratocan and lumican, were used. Furthermore, the mRNA expression of *keratocan* was quantified.

More cells in the culture cultivated without serum expressed keratocan and lumican (Supplementary Figure S2A). This confirms the hypothesis that the culture without serum allowed the growth of keratocytes. This fact is also in accordance with the literature; Mckay et al. described that exposure to low-serum conditions preserve the keratocyte phenotype [24]. 

In addition, the mRNA expression of *keratocan* was downregulated in the culture with FBS (0.30 ± 0.04; *p* = 0.04) in comparison to the one cultivated without serum (1.16 ± 0.44; Supplementary Figure S2B). This fact corroborates the hypothesis already discussed previously that the low amount of serum allows for the proliferation of keratocytes.

The cytoskeletal protein vimentin is a member of the intermediate filament family type III and is considered a marker of mesenchymal cells [9]. However, it is also reported that keratin and vimentin are co-expressed in the proliferative rim of growing colonies [9]. Zito-Abbad et al. also described that undifferentiated epithelial cells express vimentin [27]. In addition, corneal keratocytes present a basal expression of vimentin, and an increase in vimentin expression by stromal cells occurs after an injury, when myofibroblasts express vimentin [8].

To analyze the presence of mesenchymal cells and undifferentiated epithelial cells at our cultures compared to the amount of epithelial corneal cells, an antibody targeting vimentin was used. It was possible to observe that both cultures presented cells that are vimentin positive, meaning that there were mesenchymal cells and undifferentiated epithelial cells in both cultures, with and without serum (Supplementary Figure S3).

To verify the proportions of different cell types, a quantification of CK3-, lumican-, and keratocan-positive cells was performed. In cultures cultivated without FBS, an average of 80.23% epithelial cells, 19.31% keratocytes, and 0.44% of other cell types was demonstrated. Regarding cultures cultivated with 10% FBS, an average of 67.36% epithelial cells, 9.86% keratocytes, and 22.76% of other cell types was verified (Supplementary Figure S4).

## 5. Conclusions

Here, two different methods of corneal cell isolation were described, the outgrowth and the collagenase methods. In addition, the effects of fetal bovine serum were also discussed. The explants isolation for the outgrowth method (2 h) can be performed faster than the cell isolation with the collagenase protocol (5 h). Moreover, the main advantage of the collagenase method is the development of mature cells in a shorter time period. However, since there is no intact limbal region anymore, the amount of cell proliferation is limited earlier in comparison to the outgrowth method. Another disadvantage of the collagenase method is that the number of porcine corneas needed is larger than the amount needed for the outgrowth method. Two corneas are needed for one well of a 12-well plate, while only one cornea is needed for each T25 flask for the outgrowth method. Regarding the use of FBS, the cultures incubated with FBS provide a higher total amount of cells. At four weeks of cultivation, an average of 1,477,333 cells per eye was found. These cells are predominantly epithelial cells and fibroblasts, while the cell cultures without FBS provided mainly epithelial cells and keratocytes. Moreover, cell cultures without FBS provide less cells per cultivated cornea; at four weeks of cultivation, there was an average of 248,069 per eye.

However, one limitation of both the outgrowth and collagenase methods is the fact that in neither method will a pure culture of corneal epithelial cells be obtained. If a pure corneal epithelial cell culture is envisaged, there are a number of strategies that can be applied. Therefore, to increase the purity of the culture, additional purification steps can be added, such as differential trypsinization, density gradient centrifugation, or magnetic bead sorting using specific antibodies against cell surface markers [28,29,30].

On the other hand, a corneal cell culture that is not pure may have some advantages in certain experimental contexts. In vivo, corneal epithelial cells exist in close proximity to stromal cells, and the interaction between these cell types is important for maintaining corneal health. A mixed corneal cell culture may better mimic this in vivo environment and provide a more physiologically relevant model for studying corneal biology. Corneal epithelial cells and stromal cells communicate with each other through various signaling pathways. A mixed cell culture may allow for crosstalk between these cell types, potentially leading to more accurate and informative results in certain experimental contexts [31,32].

In conclusion, the outgrowth protocol can be useful to study diseases that lead to limbal insufficiency, because the corneal explants are rich in limbal epithelial stem cells (LESCs) [16]. To study wound healing, the collagenase model can be used, because once the cells are confluent, scratch assays can be performed, and the re-epithelization of these areas can be evaluated without the need of splitting the cells from the well plates. In addition, both methods can also be used to test the toxicology of new ocular drugs in vitro.

## Figures and Tables

**Table 1:** Quantity of cells.

Place	Quantity of Cells
24-well plate	100,000
96-well plate	25,000
Chamber slide	25,000–50,000

**Table 2:** Antibodies and dilutions.

Primary Antibody	Manufacturer and Dilution	Secondary Antibody	Manufacturer and Dilution
Cytokeratin-3 (epithelial cell marker)	Biorbyt (Cambridge, UK) Orb5866 1:50	AlexaFluor 488 anti rabbit	Thermo Fischer (Karlsruhe, Germany) A11008 1:500
Occludin (tight junction marker)	Santa Cruz (Heidelberg, Germany sc-133256 1:50	AlexaFluor 488 anti mouse	Thermo Fischer (Karlsruhe, Germany) A11001 1:500
Vimentin (undifferentiated epithelial cell and mesenchymal cell marker)	Sigma (Taufkirchen, Germany) V2258 1:100	AlexaFluor 555 anti mouse	Thermo Fischer (Karlsruhe, Germany) A21422 1:500
Keratocan (keratocyte marker)	Santa Cruz (Heidelberg, Germany) sc-33243 1:50	AlexaFluor anti goat	Thermo Fischer (Karlsruhe, Germany) A11055 1:500
Lumican (keratocyte marker)	Santa Cruz (Heidelberg, Germany) sc-27718 1:50	AlexaFluor anti goat	Thermo Fischer (Karlsruhe, Germany) A11055 1:500
ZO-1 (tight junction marker)	Santa Cruz (Heidelberg, Germany) sc-33725 1:50	AlexaFluor anti rat	Invitrogen (Darmstadt, Germany) 11006 1:500

**Table 3:** Primer pairs in 5′-3′ directions used for a quantitative real-time PCR.

Primer	Forward	Reverse
*GAPDH*	GGGCATGAACCATGAGAAGT	AAGCAGGGATGATGTTCTGG
*ACTB*	CACGCCATCCTGCGTCTGGA	AGCACCGTGTTGGCGTAGAG
*CK12*	TGGTCTCATCGCAAGTTCAG	TAAAGACCAACATGGCCACA
*Keratocan*	TCCCAGGGAGTGTTTCTGTC	GCATTCTCGAATGGCTTCTC

**Table 4:** Comparison between outgrowth and collagenase methods: (+) advantage and (-) disadvantage.

Outgrowth Method	Collagenase Method
+ Explants with the limbal section provide a long-term outgrowth of corneal cells.	+ Fast availability of corneal cells.
+ Cell replacement/regeneration.	+ Easier cultivation.
+ Higher number of corneal cells after a certain time period.	
- It takes 4–5 weeks for corneal cells to grow and differentiate into specific corneal cell types.	- Stem cell function is missing.
- Cell cultivation is difficult because explants have to grow on a surface in order for cells to grow out.	- No cell regeneration/replacement.
	- Higher number of porcine eyes necessary to perform this method.

## Data Availability

All data are provided within the main text. Further data inquiries are available from the authors upon reasonable request.

## Supplementary Materials

The following supporting information can be downloaded at: https://www.mdpi.com/article/10.3390/mps6030050/s1, Figure S1: Antibodies targeting CK3, occludin and ZO-1 were used to analyze the presence of CK3 and ZO-1 in corneal cell cultures with and without FBS; Figure S2: Antibodies targeting keratocan and lumican were used in the cell cultures to identify expression of these keratocyte’s markers and quantification of *keratocan* mRNA expression was performed; Figure S3: Vimentin positive cells; Figure S4: Proportion of different cell types.
