# Supplementary material for: Two Methods for the Isolation and Cultivation of Porcine Primary Corneal Cells
Source: Methods Protoc. 2023 May 12;6(3):50. doi: 10.3390/mps6030050 (PMC10204419; doi:10.3390/mps6030050)
Supplement: Supplementary file 1 [file mps-06-00050-s001.zip › mps-2255144-supplementary.pdf]

methods and protocols

MDPI

Supplementary material

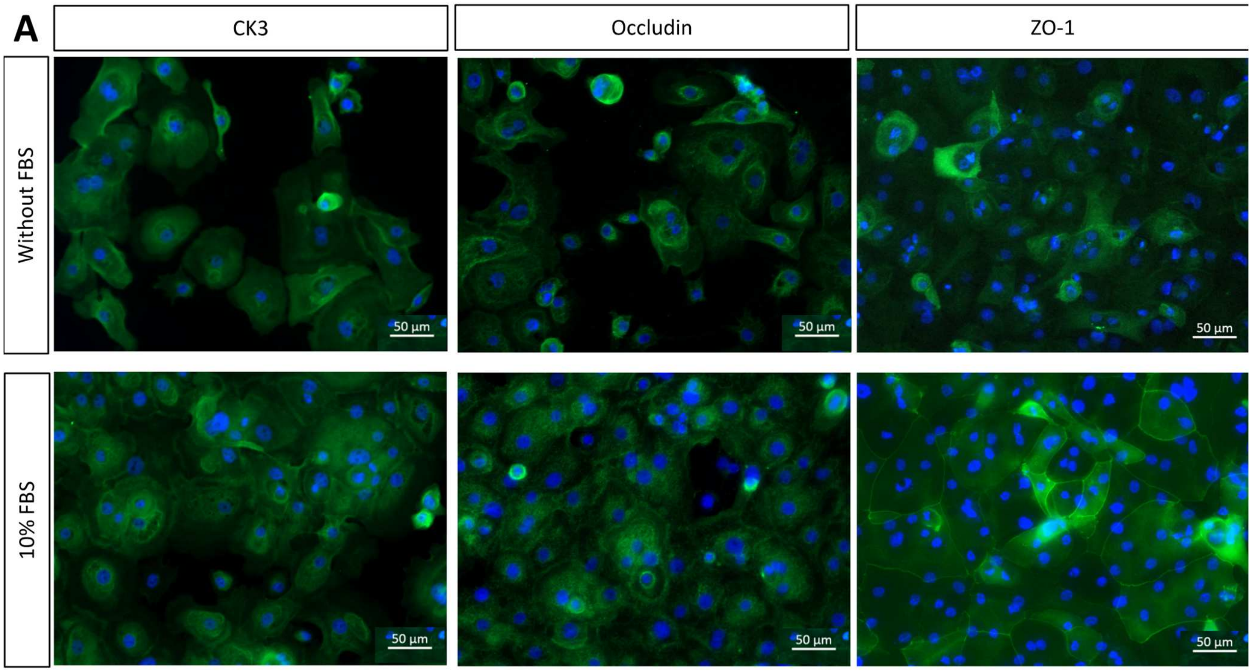

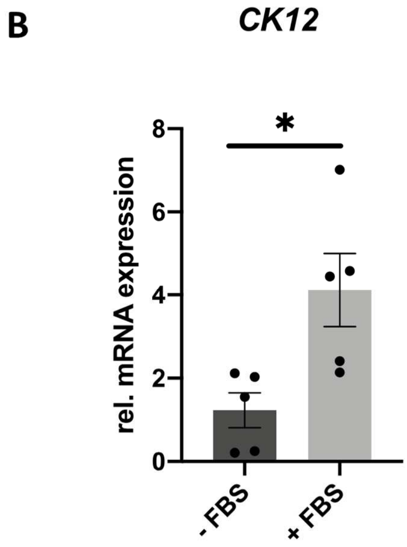

Figure S1. Antibodies targeting CK3**,** occludin and ZO-1 were used to analyze the presence of CK3 and ZO-1 in corneal cell cultures with and without FBS. In addition, the amount of *CK12*-mRNA expression was investigated. Cells were cultured for 4 weeks, split to chamber slides for immunostaining or frozen for qRT-PCR. CK3 and CK12 were used as epithelial cell markers, ZO-1 and occludin were used as tight junctions' markers. (**A**) CK3 signal was identified in both cell cultures, confirming the presence of corneal epithelial cells. Occludin and ZO-1 were also identified at both cell cultures. CK3 = green, ZO-1 = green, occludin = green, DAPI = blue. Scale bar = 50 µm. (**B**) When the gene expression of *cytokeratin 12* was compared between the cell cultures, an upregulation of *CK12* was observed at the cell culture cultivated with FBS (n = 5 for cultures without FBS and for cultures with FBS). The bars represent the mean values, and the SEM is shown. The dots represent the n number in each group. * $p < 0.05$.

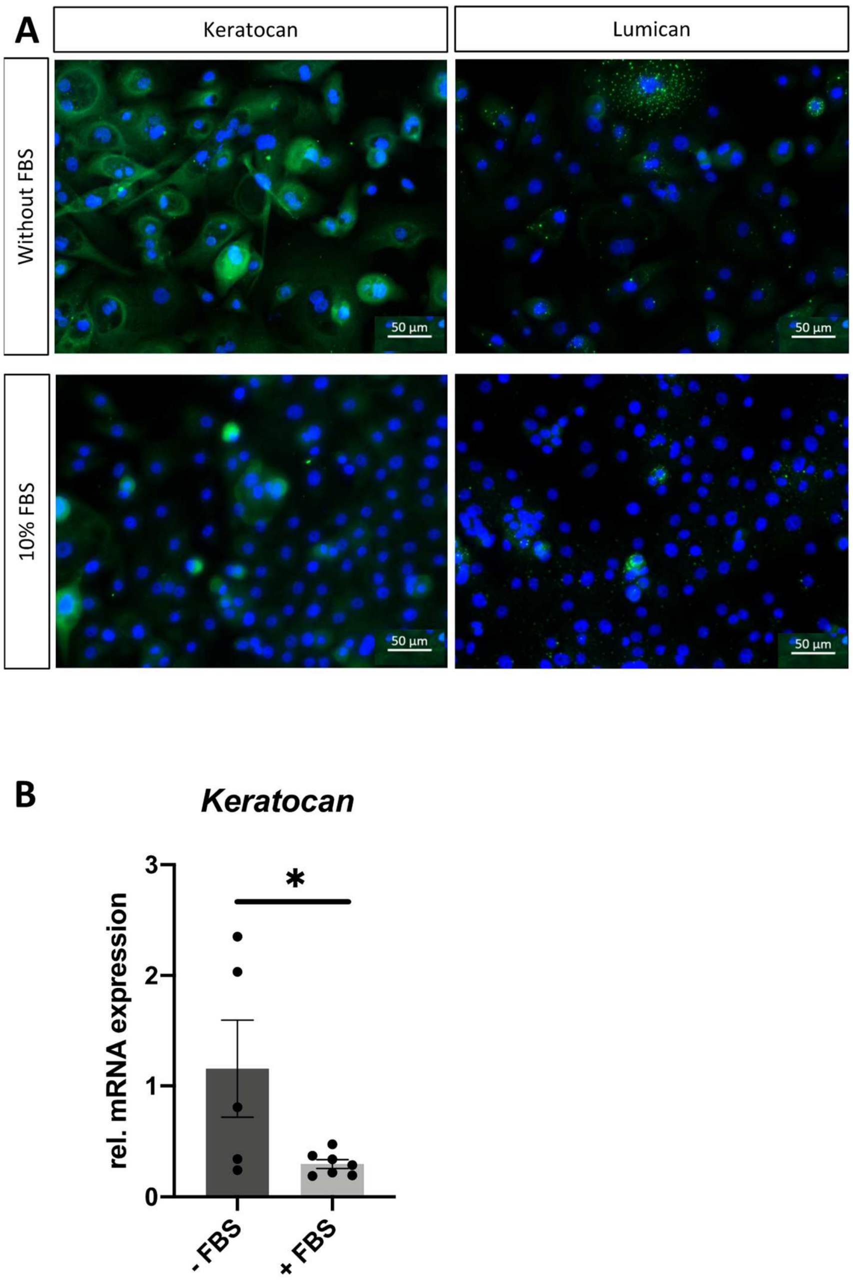

Figure S2. Antibodies targeting keratocan and lumican were used in the cell cultures to identify expression of these keratocyte's markers and quantification of *keratocan* mRNA expression was performed. (**A**) More keratocan positive and lumican positive cells were identified in the cell culture cultivated without FBS in comparison to the one cultivated with FBS, confirming a higher presence of keratocytes at the culture cultivated without FBS. Keratocan = green, Lumican = green, DAPI = blue. Scale bar = 50 µm. (**B**) A downregulation of *keratocan* was noticed in the culture with FBS when compared with the culture without FBS (n = 5 for cultures without FBS/ n = 7 for cultures with FBS). The bars represent the mean values, and the SEM is shown. The dots represent the n number in each group. * $p < 0.05$.

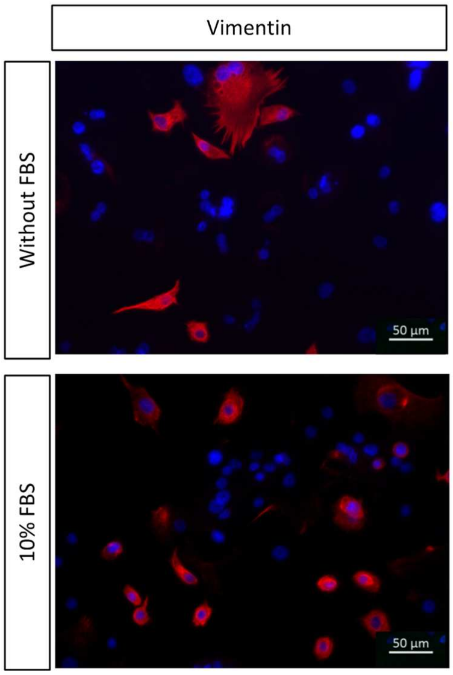

Figure S3. Vimentin positive cells. Vimentin positive cells, which are mainly mesenchymal cells and undifferentiated epithelial cells were found in both cell cultures cultivated with FBS and without it. Vimentin = red, DAPI = blue. Scale bar = 50 µm.

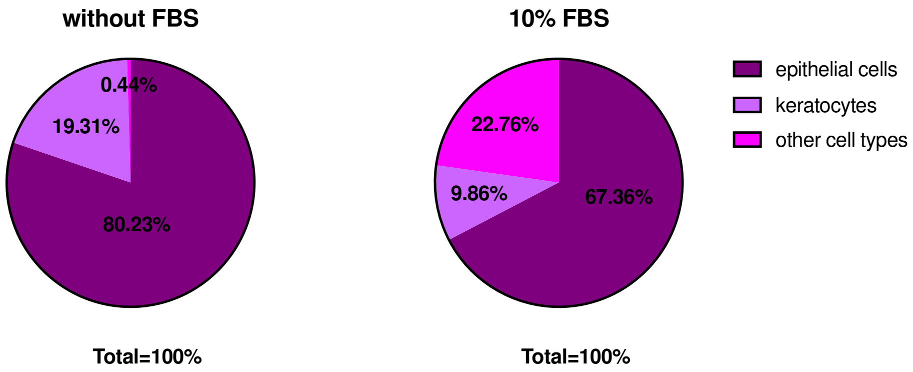

Figure S4. Proportion of different cell types. Cultures cultivated without FBS presented a majority of epithelial cells, some keratocytes and a small proportion of other cell types. In cultures cultivated without FBS the majority was again of epithelial cells, but other cell types had a bigger proportion than keratocytes.
